# Two Old-Fashioned Remedies in Gout

**Published:** 1900-06-30

**Authors:** 


### TWO OLD-FASHIONED REMEDIES IN GOUT.

In the treatment of chronic gout, Dr. James. Edmunds[1] recommends two old-fashioned remedies, which, although we have no doubt they are constantly employed by many practitioners, are apt to be crowded out of the minds of the younger generation by the newer preparations which are introduced every year We allude to bitartrate of potash as an alkali, and a mixture of this salt with guaiacum and sulphur as an aperient. For non-febrile chronic cases 20 grains of potassium bitartrate may be given twice or three times a day either dissolved in Salutaris or other pure aërated distilled water, or in hot water, which has been boiled to throw down the chalk, and remove other possible impurities. With persons of spare habit a bit of sugar and a flavour of lemon peel may be added. This beverage should be drunk hot like tea, and its use rapidly clears the system of urates. Potassium bitartrate taken in this way gives a pleasant beverage which is acidulous, and therefore goes well with the gastric juice during digestion, and in the tissues its tartaric acid is oxidised into $CO_2$ and $H_2O$, while nascent potassium bicarbonate is set free in a state of perfect diffusion and almost infinitesimal dilution. Its use is, therefore, quite a different thing from the projection into the stomach of a crude alkali. It may be pointed out that 29 grains of potassium bitartrate contain five grains of anhydrous potash. As for the aperient, it consists of an ounce each of pulv. guaiaci, potass. bitart., and sulphur precip., with two drachms of pulv. tragac. co. made into a powder, of which a teaspoonful may be taken at bedtime in a wineglassful of water.

[1] Brit. Med. Jour., June 9.

