# Erratum

**Published:** 1900-07-07

**Authors:** 


### ERRATUM.

In our abstract of Dr. Edmunds' note on Gout, at page 220, column 1, line 27, the words "29 grains of potassium bitartrate contain 5 grains of anhydrous potash" should have read 20 grains contain 5, &c.

